# Cervical vestibular evoked myogenic potentials and video head impulse test in Ménière disease^☆^

**DOI:** 10.1016/j.bjorl.2019.02.002

**Published:** 2019-03-16

**Authors:** Thaís Alvares de Abreu e Silva Grigol, Karen de Carvalho Lopes, Fernando Freitas Ganança

**Affiliations:** Universidade Federal de São Paulo (Unifesp), Escola Paulista de Medicina (EPM), Disciplina de Otologia e Otoneurologia, São Paulo, SP, Brazil

**Keywords:** Head impulse test, Ménière disease, Vestibular evoked myogenic potentials, Vestibular function tests, Teste do impulso de cabeça, Doença de Ménière, Potenciais evocados miogênicos vestibulares, Testes de função vestibular

## Abstract

**Introduction:**

Ménière's disease is among the most frequent causes of vestibular disorders. Although it is a clinical diagnosis, a better understanding of the pathophysiology and clinical course of the disease through tests would allow improvement in the prognosis and more effective treatments.

**Objectives:**

To describe the results of the cervical vestibular evoked myogenic and video head impulse test in patients with a defined diagnosis of Ménière's disease and to correlate them with disease duration.

**Methods:**

The sample consisted of 50 participants, of whom 29 comprised the study group and 21 the control group. The individuals were submitted to a questionnaire, otoscopy, audiometry and vestibular function assessment through the cervical vestibular evoked myogenic potential and video head impulse test.

**Results:**

For the video head impulse test, lateral canal gain values below 0.77 were considered abnormal and for the vertical channels, below 0.61. The percentages of normality were 82.76% for lateral, 89.65% for posterior and 91.37% for anterior canals. For the cervical vestibular evoked myogenic potential, the upper limits of normal for latencies were defined as 18.07 ms for p13 and 28.47 ms for n23; and in the SG, 19.57% showed prolongation of latency of p13 and 4.35% of wave n23, whereas 18.96% did not show biphasic potential.

**Conclusions:**

For the video head impulse test, a decreased gain of the vestibulo-ocular reflex for the lateral canal was observed, with a higher incidence of overt type corrective saccades compared to the control group. For the cervical vestibular evoked myogenic potential, there was a significant difference between the groups for the inter-amplitude parameter, including for asymptomatic ears. There was no correlation between the results of the tests and disease duration.

## Resumo

**Introdução:**

A doença de Ménière está entre as causas mais frequentes de vestibulopatias. Apesar de o diagnóstico ser clínico, compreender melhor a fisiopatologia e o curso clínico da doença por meio dos exames vestibulares permite melhores prognósticos e tratamentos.

**Objetivos:**

Descrever resultados do potencial evocado miogênico vestibular cervical e teste de impulso cefálico por vídeo em pacientes com diagnóstico de doença de Ménière definida e correlacionar com o tempo de doença.

**Método:**

A amostra foi constituída por 50 participantes, dos quais 29 compuseram o grupo de estudo e 21 formaram o grupo controle. Os indivíduos foram submetidos a um questionário clínico, otoscopia, avaliação audiológica e avaliação da função vestibular por meio do potencial evocado miogênico vestibular cervical e teste de impulso cefálico por vídeo.

**Resultados:**

Para teste de impulso cefálico por vídeo foram considerados alterados os valores de ganho para canal lateral abaixo de 0,77 e para os canais verticais abaixo de 0,61; e os percentuais de normalidade para o grupo de estudo foram 82,76% para lateral; 89,65% para posterior e 91,37% anterior. No potencial evocado miogênico vestibular cervical, os limites superiores das latências foram definidos 18,07 ms para p13 e 28,47 ms para n23; no grupo de estudo 19,57% apresentaram prolongamento da latência da p13 e 4,35% da onda n23 e 18,96% não apresentaram o potencial bifásico.

**Conclusões:**

No teste de impulso cefálico por vídeo observou-se ganho do reflexo vestíbulo ocular diminuído para os canais laterais, com maior ocorrência de sacadas corretivas do tipo *overt*. Para o potencial evocado miogênico vestibular cervical observou-se diferença significante entre os grupos para o parâmetro interamplitude, inclusive para orelhas assintomáticas. Não foi evidenciada correlação dos resultados dos exames com o tempo de doença.

## Introduction

Ménière's disease (MD), initially described in 1861, is one of the most frequent vestibular disorders. It is considered a multifactorial disease, characterized by symptoms of episodic vertigo, fluctuating hearing loss, tinnitus and aural fullness.^1^, ^2^

Endolymphatic hydrops, the distention of the endolymphatic space, was discovered by Hallpike and Cairns^3^ in the temporal bones of patients with this condition. It has long been believed that endolymphatic hydrops alone is the histopathological substrate of MD, but this finding cannot explain the complexity of the clinical picture.

The diagnosis of MD is clinical and based on the presence of characteristic symptoms. According to the current classification, published in 2015, released by the Bárány Society, The Japan Society for Equilibrium Research, the European Academy of Otology and Neurotology (EAONO), the American Academy of Otolaryngology-Head and Neck Surgery (AAO-HNS) and the Korean Balance Society committees, MD can be classified as Definite or Probable. MD is categorized as Definite when there are two or more episodes of vertigo lasting between 20 min and 12 h, documented sensorineural loss at low or medium frequency (below 2 kHz) and symptoms of hearing fluctuation (hearing loss, tinnitus and/or aural fullness). The Probable MD is characterized by two or more episodes of vertigo lasting between 20 min and 24 h and symptoms of hearing fluctuation (hypoacusis, tinnitus and/or aural fullness).^1^

According to temporal bone studies, hydrops formation occurs in the cochlea, sacculus, utricle and semicircular canals, in a decreasing order of frequency.^4^, ^5^ Although the diagnosis is a clinical one, a better understanding of the pathophysiology and clinical course of the disease could result in a more accurate and timely diagnosis and lessen the impairment of the quality of life of patients with MD. Therefore, the exams that assess the function of the auditory and vestibular system could assist in the treatment, especially in the early stages.^6^ Currently, because of the high prevalence of hydrops in the saccular region, one of the more frequently utilized exams is the cervical Vestibular Evoked Myogenic Potential (cVEMP). cVEMP is a short-latency, biphasic muscle potential that evaluates the inferior portion of the vestibular system, specifically the saccule, inferior vestibular nerve, vestibular-spinal pathways and neuromuscular plaque. After auditory stimulation, the vestibular-spinal muscle reflex is manifest by a contraction of the cervical musculature.^7^, ^8^

It was observed that cVEMP in MD is very useful, as it helps to detect and document changes in these vestibular regions and to identify the disease stage. The literature findings are diverse, and an absence of biphasic potentials, increased latency, decreased inter-amplitude interval and increased asymmetry index have all been described in MD.^9^, ^10^, ^11^ Another interesting finding is that of the presence of abnormalities in the asymptomatic ear, such as absence of certain waves or increased latency, which may reveal the possible evolution of the disease as occult bilateral MD.^12^, ^13^

Another very current exam that allows the detection of alterations in the Oculovestibular Reflex (OVR) in the three semicircular canals is the Video Head Impulse Test (vHIT). It identifyies and quantifies the Head Impulse Test (HIT) test commonly performed in neurotological evaluation.

During the evaluation, rapid and unpredictable head movements are applied in the horizontal and vertical planes and the eyes must remain at a fixed point. When there is an abnormal OVR, the eyes do not remain fixed and there are ocular compensatory movements, called saccades. The saccades can be triggered during the cephalic impulse (covert), not visible to the naked eye, or after the impulse (overt).^14^ Several parameters such as OVR gain, and saccade characteristics (latency, velocity, and rate of occurrence) gave the vHIT increased sensitivity and specificity when compared to the HIT.^15^

The literature findings on the use of vHIT in MD are variable. Most studies show normal gain or small reduction in OVR gain.^16^, ^17^, ^18^ Some studies correlate the results obtained at this exam with the evaluated period (crisis or inter-crisis), time of disease and degree of hearing loss.^19^, ^20^

The cVEMP and vHIT are easy to perform, non-invasive, and well tolerated by the patient. They both allow a functional evaluation of the vestibular system, which favors the early investigation and monitoring of the clinical evolution of MD. Therefore, objective and reliable tests such as these can help in the differential diagnosis.

This study was proposed after noting the high prevalence of MD in the population and the need for complementary tests that investigate and help in the disease monitoring and evolution. Few studies have evaluated MD according to the current classification of 2015. Thus, we seek to increase knowledge by exploring and identifying the structures involved in MD and the effects on body balance, which may contribute to the choice of the best therapeutic strategy.

The objectives of this study were to evaluate the results of the cVEMP and vHIT in patients diagnosed with definite MD in the symptomatic and asymptomatic ears and to correlate test results with the time of disease.

## Methods

This is an observational, cross-sectional study carried out at the Department of Otorhinolaryngology and Head and Neck Surgery of Escola Paulista de Medicina/Universidade Federal de São Paulo, after approval by the Research Ethics Committee, number 706.875/2014.

The sample consisted of 50 participants, of whom 29 constituted the Study Group (SG) and 21 the Control Group (CG). Patients of both genders aged between 18 and 70 years, with a medical diagnosis of definitive unilateral or bilateral Ménière's disease according to the classification of the current Committees (2015), were included in the SG.^1^ This clinical diagnosis was considered as the gold standard in this study.

The CG consisted of volunteers without complaints or history of dizziness, hearing loss, otologic surgery or middle ear abnormalities.

All study participants received explanations and information on the study and were asked to sign the Free and Informed Consent Form. The following were excluded from the study: patients with probable MD, patients who had undergone some invasive procedure, history of head trauma, otologic surgery, middle ear and retrocochlear diseases, neurological and psychiatric diseases, cervical rotation limitations and visual impairment.

After the clinical evaluation, performed by the same otorhinolaryngologist, all subjects were submitted to a clinical-demographic questionnaire, otoscopy, audiological and vestibular function evaluation with cVEMP and vHIT.

The disease was stageed according to the AAO-HNS Auditory and Balance Committee (1995) classification, based on the mean of the tonal thresholds of 500 Hz, 1 kHz, 2 kHz and 3 kHz, in the symptomatic ear, at the worst audiometry. This mean subdivides the disease stage into Stages I (≤25 dB), II (26–40 dB), III (41–70 dB) and IV (>70 dB).

The ICS Charp EP 200 (GN-Otometrics) equipment was used to perform the cVEMP, Disposable surface electrodes were used, after skin dermabrasion, to capture responses and control the Electromyographic (EMG) activity. The maximum impedance allowed for the electrodes was 5 ohms (Ω). The placement of the surface electrodes followed this order: active electrode placed in the upper half of the sternocleidomastoid muscle, ipsilateral to the sound stimulation and below the electrode that controls the electromyographic activity; the reference electrode, on the manubrium of the sternum bone and the ground electrode in the frontal midline. The patient remained seated, with maximal cervical rotation of the head to the side contralateral to the stimulus.^21^

The rarefaction tone burst stimuli, presented through insertion phones, with a total of 150 stimuli, was used for cVEMP recording. The tested frequency was 500 Hz, with a 97 dBHL intensity, 10 Hz high-pass and 1000 Hz low pass filters, and 60 ms recording window.

**Figure 1:**
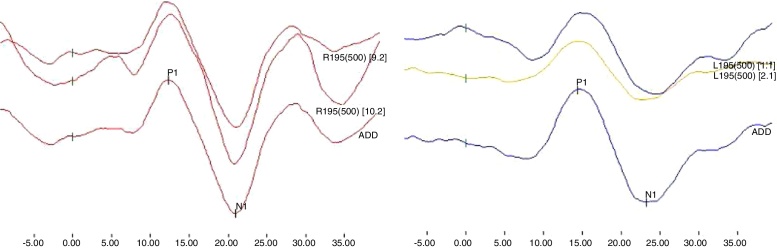
Example of Cervical Vestibular Evoked Myogenic Potential evoked bilaterally.

The acquired responses consisted of a biphasic potential with the first positive wave exhibiting a latency of approximately 13 ms, known as p13, and the second negative wave having a latency of approximately 23 ms, known as n23. Each ear was tested until reproducible responses were obtained, and the two best waves were chosen, with the weighted sum of both being performed and a third record was obtained, which was used for analysis (Fig. 1). Electromyographic responses were accepted between 50 and 200 μV, in order to maintain adequate and constant contraction level. The cVEMP parameters considered in this study were the presence of biphasic potential, absolute latencies of p13 and n23, p13–n23 inter-amplitude interval and the Asymmetry Index (AI).

The interpretation of the cVEMP results was based on the reference values determined by the CG. The reference values for the interpretation of normality for p13 and n23 latencies, p13 and n23 inter-amplitude and AI were calculated by the mean ± 2 standard deviations (SD).^22^ This method was selected to allow the qualitative classification of the results of cVEMP as normal or altered, unilateral or bilateral. The absence of the biphasic potential, an increase in the latency of p13 or n23, a decrease in the p13–n23 inter-amplitude and the AI above the upper limit were interpreted as abnormal responses.

The ICS Impulse (GN-Otometrics) equipment was used to perform the vHIT. The vHIT system consists of glasses and integrated video-oculography camera with sensors that are connected to the head by an elastic band. The camera analyzes the movement of the eyes at a sampling rate of up to 250 Hz and has a mirror that reflects the image of the patient's eye into the camera. A small sensor in the glasses measures the head movement. The test was applied with the individual sitting in a chair approximately 1.0 m away from the target, during which the subject is instructed to keep the eyes on a fixed point.^19^, ^23^

The researcher applied unpredictable frequency and direction movements in the planes of the semicircular canals, with low amplitude (near 10° to 20°), high velocities (above 100° s) and accelerations (2000–6000 s^2^). First, movements were performed in the horizontal plane to evaluate the lateral canals. Soon after, the patient's head was turned 30° to the right, and head movements were performed back and forth in the vertical plane to test the synergic pairs of the left anterior and right posterior (LARP – Left anterior and Right Posterior) semicircular canals. Subsequently, the instruction was repeated with the head at 30° to the left and the right anterior and left posterior (RALP – Right Anterior and Left Posterior) canals were evaluated. In all evaluations, the individual was instructed to keep the eyes on a fixed point. During the test, at least 10 head impulses were performed in each evaluated plane.^14^, ^19^, ^24^

**Figure 2:**
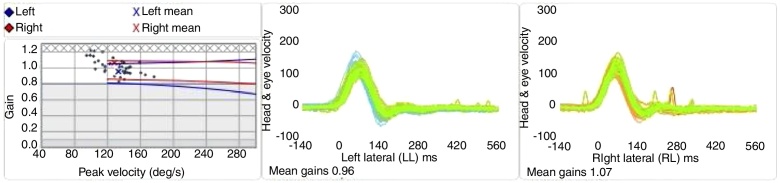
Example of the video head impulse test recording in the lateral canals.

Based on the recording of the cephalic and ocular velocity, the calculation of the OVR gain was performed by the equipment. The OVR gain was represented by the mean ratio of these velocities (Fig. 2). The corrective saccades were detected by the equipment and qualitatively classified according to their latencies. The covert saccades occurred before the end of the impulse, approximately 100 ms and the overt saccades 100 ms after the head movement.^14^, ^25^

The interpretation of the vHIT results was based on the reference values determined by the CG. The vHIT results were calculated by the mean ± 2 SD. Abnormal responses were decreased OVR gain and the presence of corrective saccades (covert or overt).

The data were tabulated and analyzed in order to describe the clinical and demographic characteristics of patients and to establish a pattern of distribution between the groups. The Shapiro–Wilks test was used to evaluate the normal distribution between the groups. Parametric tests were used when there was a normal distribution, and non-parametric ones when there was a non-normal distribution.

The effect size of the difference between the groups for each value was measured by calculating the coefficient *d* or the coefficient *r*. In the correlation analysis, the correlation coefficient (*r*) was calculated, which could vary from −1 to +1. The statistical significance was set at 5% (*p* < 0.05). The 95% Confidence Intervals (95%CI) were calculated using the bias-corrected and accelerated method, based on 2000 bootstrap samples.

## Results

The study sample consisted of 50 individuals, distributed into two groups. The Study Group (SG) consisted of 18 women and 11 men, with a mean age of 52.24 years and the Control Group (CG) of 15 women and 6 men, with a mean age of 39.67 years.

As for the sample homogeneity study, no statistically significant differences were observed between the groups in relation to gender (*p* = 0.557). Regarding age, the groups did not show a normal distribution. The results showed there was a statistically significant difference between the groups, with the patients from the SG being older than those from the CG.

Regarding disease laterality, 22 (75.9%) patients had unilateral MD and 7 (24.1%) had bilateral MD, totaling 36 affected ears in the sample. As for the distribution of unilateral MD, 12 (41.4%) had MD in the right ear and 10 (34.5%) in the left ear. Of the patients with bilateral MD, all had the initial symptoms in only one ear and, subsequently, the disease progressed to the opposite ear.

The time of disease evolution until the contralateral ear was affected ranged from 2 months to 15 years. The median duration of the disease was 6 years, with a mean 8.57 years, ranging from 4 months to 25 years.

The initial symptoms, disease stage (hearing loss in the ear with MD) and the presence of headache (migraine or non-migraine) are shown in Table 1.

**Table 1:** Clinical characteristics of patients with Ménière's disease.

Initial symptoms	Stage of disease	Headache
Dizziness: 21 (72.41%)	I: 7 (18.92%)	Non-migraine: 9 (31.03%)
Tinnitus: 18 (62.07%)	II: 11 (29.73%)	
Hearing loss: 14 (48.28%)	III: 17 (45.95%)	Migraine: 5 (17.24%)
Aural fullness: 8 (27.59)	IV: 2 (5.40%)	

To calculatethe the normality pattern for vHIT, 2DP were subtracted from the mean gain values. Therefore, gain values for lateral canal <0.77 and gain values for vertical channels <0.61 were considered abnormal. In the analysis of the gain values for OVR in the SG, with respect to the symptomatic and asymptomatic ears (58 ears), 82.76% (48 ears) had normal results for the lateral canal, 89.65% (52 ears) for the posterior canal and 91.37% (53 ears) for the anterior canal.

Eleven ears in the SG (18.96%) did not show a biphasic potential response nor did 3 ears (7.14%) in the CG. For the analysis of cVEMP parameters, subjects with no response were excluded, so the SG consisted of 25 ears and the CG of 39 ears.

The asymmetry index (AI), a parameter calculated only in the presence of bilateral response, was obtained in 19 individuals from the SG and 19 from the CG. The upper limits of normal for the latencies were defined by the mean plus 2 SD: 18.07 ms for p13 and 28.47 ms for n23. It was observed that 19.57% of the ears in the SG showed prolongation of the p13 latency and 4.35% of the n23 wave.

According to the ear affected by MD, the SG was subdivided into symptomatic (*n* = 36) and asymptomatic (*n* = 22) ears.

There was a statistically significant difference among the three groups regarding lateral canal gain (*p* = 0.002), and post hoc analysis showed a difference between symptomatic ears and the CG, but not between the symptomatic and asymptomatic ears (*p* = 0.718) and between the asymptomatic ears and the CG (*p* = 0.093). The symptomatic ears showed less gain in the lateral canal in the vHIT compared to the ears of the CG. Regarding the gain of the posterior and anterior canals, there was no difference between the ears (Table 2).

**Table 2:** Descriptive values and comparative analysis of the ears in relation to the vestibulo-ocular reflex gain values, according to the evaluated canal, at the video head impulse test.

Gain	Ear	Mean	SD	Median	Min	Max	*p*	Post hoc	*p*	*d*
Lateral canal	Symptomatic	0.89 [0.84/0.95]	0.17	0.93 [0.89/0.95]	0.59	1.26	0.002^a^	S × A	0.718	0.303
	Asymptomatic	0.93 [0.88/0.86]	0.12	0.92 [0.85/1.01]	0.73	1.13		S × C	0.001^a^	0.935
	Control	1.01 [0.97/1.05]	0.12	1.02 [0.96/1.06]	0.76	1.24		A × C	0.093	0.645
Posterior canal	Symptomatic	0.82 [0.76/0.87]	1.81	0.82 [0.76/0.88]	0.27	1.31	0.653	Does not apply	–	–
	Asymptomatic	0.79 [0.73/0.86]	0.18	0.82 [0.75/0.86]	0.37	1.18				
	Control	0.83 [0.80/0.86]	0.11	0.85 [0.85/0.85]	0.52	1.08				
Anterior canal	Symptomatic	0.80 [0.74/0.86]	0.18	0.76 [0.72/0.86]	0.41	1.17	0.286	Does not apply	–	–
	Asymptomatic	0.84 [0.77/0.92]	0.18	0.83 [0.74/0.92]	0.48	1.20				
	Control	0.86 [0.82/0.90]	0.13	0.87 [0.83/0.91]	0.60	1.16				

*Note*: One-way ANOVA with post hoc analysis using the Gabriel test.

SD, standard deviation; Min, minimum; Max, maximum; *d*, effect size.

The values in brackets indicate the upper and lower limits of the 95% Confidence Intervals.

a Statistically significant value at the 5% level (*p* < 0.05).

When considering the presence of corrective saccades in vHIT, there were no corrective saccades in the posterior and anterior canals in both groups. When comparing the ears in relation to the occurrence of saccades in the lateral canal, a significant difference was observed between the groups of ears. Saccades were absent more often in the asymptomatic ears of theb SG than in the CG. Regarding the saccade subtypes (overt and covert), there was no statistical significance, despite the more frequent presence of overt lateral saccades.

Regarding the presence of the biphasic potential of cVEMP, there was a statistically significant difference between the groups. Failure to record the potential in the symptomatic ears was more common than such a failure to record it in the asymptomatic ears and the CG. Regarding the asymptomatic ears and controls, there was no difference in relation to the presence of cVEMP.

For the analysis of cVEMP results, the values of each ear were considered individually, so it was not possible to establish this comparative analysis for AI. The results of Table 3 show that there was a statistically significant difference among the three groups regarding cVEMP inter-amplitude (*p* < 0.002), and the post hoc analysis showed that there was a statistically significant difference between the group of symptomatic ears and the CG (*p* < 0.001), and between the group of asymptomatic ears and the CG (*p* = 0.001). Thus, both symptomatic and asymptomatic ears of individuals with MD showed lower cVEMP inter-amplitude when compared to the CG. There was no statistically significant difference in the latency values of waves p13 and n23 between the ears (Table 3).

**Table 3:** Descriptive values and comparative analysis of the ears in relation to the parameters evaluated by the Cervical Vestibular Evoked Myogenic Potential in the groups of symptomatic, asymptomatic and control ears.

cVEMP	Ear	Mean	SD	Median	Min	Max	*p*	post hoc	*p*	*d*
Latency p13 (ms)	S	15.40 [14.37/16.56]	2.80	14.33 [13.95/15.83]	12.25	21.50	0.134^a^	Does not apply	–	–
	A	14.34 [13.37/15.43]	2.41	13.93 [12.75/14.67]	11.83	19.83				
	C	14.99 [14.51/15.46]	1.54	15.00 [14.75/15.33]	11.50	18.67				
Latency n23 (ms)	S	23.70 [22.19/25.25]	3.27	23.30 [21.50/24.67]	19.00	30.60	0.074^b^	Does not apply	–	–
	A	22.52 [21.40/23.67]	2.86	22.00 [20.42/23.08]	18.33	27.67				
	C	24.22 [23. 53/24.91]	2.13	24.67 [23.67/25.00]	19.42	28.50				
Inter-amplitude (μV)	S	86.80 [68.57/105.62]	51.90	72.05 [68.48/81.62]	18.68	213.47	<0.001^a,c^	S × A	>0.999	0.132
	A	119.31 [84.45. 160.52]	102.22	79.51 [63.08/114.58]	17.72	445.86		S × C	<0.001^a^	0.602
	C	215.05 [176.20/258.90]	135.82	174.56 [135.03/208.10]	56.38	640.70		A × C	0.001^a^	0.462

*Note*: Kruskal–Wallis test (a) with post hoc analysis using the Dunn–Bonferroni test and one-way ANOVA (b).

cVEMP, Cervical Vestibular Evoked Myogenic Potential; S, symptomatic; A, asymptomatic; C, control; SD, standard deviation; Min, minimum; max, maximum; *d*, effect size.

The values in brackets indicate the upper and lower limits of the 95% confidence intervals.

^c^ Statistically significant value at the 5% level (*p* < 0.05).

Attempting to ascertain whether there was a correlation between time of the disease and the performed tests, *p* value was calculated using Spearman's (non-parametric) or Pearson's (parametric) tests, and the correlation coefficient (*r*). The vHIT parameters showed no statistically significant correlation with time of the disease (Table 4).

**Table 4:** Correlation analysis between time of disease and exam parameters: Cervical Vestibular Evoked Myogenic Potentials and video head impulse test for the symptomatic ears.

Variable		Time of disease
cVEMP – Latency P13	*r*	−0.059 [−0.423/0.409]
	*p*	0.780^a^
cVEMP – Latency N23	*r*	−0.048 [−0.406/0.422]
	*p*	0.821^a^
cVEMP – Amplitude	*r*	0.084 [−0.190/0.403]
	*p*	0.690^a^
cVEMP – Asymmetry Index	*r*	0.054 [−0.358/0.542]
	*p*	0.826
VHIT – Lateral Gain	*r*	−0.007 [−0.368/0.331]
	*p*	0.968^a^
VHIT – Posterior Gain	*r*	−0.049 [−0.367/0.234]
	*p*	0.778^a^
VHIT – Anterior Gain	*r*	0.127 [−0.295/0.545]
	*p*	0.462^a^

*Note*: Pearson's correlation test (a) and Spearman's correlation test (b)^.^

## Discussion

With respect to the demographic characteristics of patients with MD, the sample had a higher prevalence of females (62.02%). In a study evaluating the prevalence of MD in the population, it was observed that women are more affected than men, at a ratio of 1.89:1.^26^ The mean age of the subjects was 52 years, with an age range varying from 19 to 69 years. A similar mean age was observed in another study,^27^ and a similar age range has also been reported.^4^, ^28^, ^29^, ^30^ As for the mean age of disease onset, the current study found a mean of 44.1 years, a result similar to that observed in other studies.^31^, ^32^

In the present study sample, most patients had unilateral disease 22 (75.90%) and of these, the right ear was affected in 12 subjects (54.50%). Most studies included patients with unilateral MD^12^, ^18^, ^19^, ^27^ and the predilection for one of the ears was not a consensus.

Considering the prevalence of bilateral MD (24.10%), another study also obtained a similar prevalence, with 20.00% of the patients diagnosed with involvement in both ears.^33^ Patients with bilateral involvement in this study initially had definite MD in one of the ears and then the disease progressed bilaterally, with time of disease evolution varying from 2 months to 15 years. In the study by Perez et al.,^34^ the time interval between the initial diagnosis and the disease onset in the opposite ear ranged from 3 to 26 years. The occurrence of bilateral involvement may indicate disease progression and depends on factors such as time of evolution and diagnostic criteria used. Bilateral MD rarely presents with simultaneous onset in both ears, but is usually sequential with the contralateral ear becoming involved only after some years.^35^

The present study showed great variation regarding the duration of disease (4 months to 25 years). This wide variation was also reported in other studies.^18^, ^27^, ^36^ As for the initial symptoms to appear in this study, dizziness and tinnitus were the most common. In agreement with the literature, the prevalence of dizziness was found to be the initial symptom, followed by tinnitus.^29^, ^37^ What may explain the high occurrence of dizziness as the initial symptom is the possibility that vestibular MD is the initial form of disease development, which is corroborated by a study that evaluated the onset of vertigo, hearing loss and tinnitus in MD and associated with endolymphatic hydrops, assessed through magnetic resonance imaging.^29^

The presence of headache with migraine characteristics was also observed in this study. The occurrence of migraine in MD can vary from 22% to 56%, being more prevalent than in the general population.^38^, ^39^ A study suggests that there may be the same pathophysiology and some genetic component between MD and migraine.^39^ Although not yet proven, one hypothesis is that vasospastic events associated with migraine result in damage to the inner ear, predisposing the ear to auditory and vestibular symptoms,^35^ as well as the presence of mutations in genes related to voltage-dependent neuronal channels.^40^

As for the degree of hearing loss, there was a prevalence in descending order of Stages III, II, I and IV. Kim et al.^37^ when studying MD, found the same proportion of prevalence of Stages II and III. Lee et al.^20^ when evaluating patients during the crisis found a higher occurrence of Stage I, then III and II. The study by Rubin et al.,^18^ which analyzed advanced unilateral MD with a progression >1 year and more than one crisis per month, found a higher proportion of Stage III, followed by IV, II and I.

MD is a multifactorial condition with a variable disease course and duration. Some authors perceive it as a continuum of an initial disease that evolves into a fully developed disease entity.^29^ However, it is a condition that is still well studied, and the tests become allies for the functional evaluation of these patients and the differential diagnosis of the disease.^5^ The prevalence of hydrops is 100% in the cochlea, 86.3% in the saccule, 50% in the utricle and 36.4% in the semicircular canals.^41^

Currently, vHIT has become a useful clinical tool to quantitatively detect the function of the three pairs of semicircular canals, through the evaluation of angular OVR.^42^

There are studies that have reported different results than those of the present study. The only study that evaluated vHIT in definite MD subjects using the same diagnostic criteria found vHIT gain to be 100% of the normal, and the established standard of normality was 0.78 for the lateral canals and 0.64 for vertical canals However, the sample included only advanced MD.^18^ Other studies using the previous AAO-HNS criteria found different results.^17^, ^20^ The discrepancies in results between the studies may be related to the differences in the methods and diagnostic criteria used, period evaluated (crisis or inter-crisis) and standard of normality for OVR gain.

Following the order of MD involvement, the semicircular canals are the last sites to be affected in the inner ear, which could explain the usually normal results in vHIT.^5^ The semicircular canals are more resistant to hydropic expansion and have thicker and more rigid walls than the saccule, for instance.^43^ Additionally, it is suggested that the dissociation between normal vHIT and altered caloric test may be an indicator that the patient has MD, since hydrops has little effect on the responses of the cupula in the cephalic impulse.^44^

An explanation for finding more abnormal results in the lateral canal, compared to vertical canals could reflect the vestibular anatomy and disease pathophysiology. The vertical canals have greater marginal space in comparison with the horizontal ones, favoring an additional resistance to the volume caused by the hydrops. Thus, the pressure that impairs the endolymphatic flow would be proportionally smaller in the vertical canals.^19^

As for the presence of saccades, the study by Blödow et al.^25^ showed a higher prevalence of the overt compared to the covert subtypes, as did the present study. This same study indicated that the occurrence of saccades in vestibular dysfunctions is common, with overt saccades appearing more frequently, alone or in combination with covert saccades, mainly in acute vestibular injury.^14^ There is a decrease in the OVR gain in vestibular loss and refixation saccades are attempt to compensate for this failure. When the patient cannot keep his eyes on the target, covert saccades occur as part of a dynamic compensation, which aim to stabilize the gaze. However, when the eyes cannot reach the target, the secondary overt saccades may appear, a clear sign of vestibular dysfunction.^25^, ^45^ As overt saccades occur later, they are related to a central visual firing mechanism; the mechanism for the covert saccades remains uncertain.^25^ It was also observed that, as cephalic impulse accelerations increased, covert saccades became more common.^45^ This finding confirms the importance of vHIT at high accelerations. Moreover, saccades are important for the understanding of plasticity during the recovery from unilateral vestibular loss, since they are related to a compensation mechanism or substitution within the oculomotor system.^46^

Supporting the results of the present study, most studies did not find vHIT to be an exam that detects changes in asymptomatic ears as a predictor of bilateral MD. In a study in which OVR was evaluated in patients after parenteral antibiotic therapy with gentamicin, a reduction in gain was also observed on the asymptomatic side, but this effect was only present for lower gain values, of around 0.38.^47^

The cVEMP, in turn, is also a broadly used clinical examination to investigate different neurological and neurotological disorders.^21^ The last systematic review carried out by the American Academy of Neurology^8^ indicated that animal studies suggest that cVEMP is more closely related to saccule function; however, the accuracy of both cervical and ocular VEMP to accurately identify the vestibular function of the saccule and utricle is still unknown. According to this review, studies indicate the possibility that VEMP can help evaluate the disease, but it is not conclusive that VEMP can be used to diagnose MD. But, this test may be useful in the clinical monitoring of patients with MD.

In the literature, most studies used the diagnostic criteria of AAO-HNS (1995) for the diagnosis of MD, but had variable and divergent results.^4^, ^9^, ^12^

The only study we found that used the current criteria of 2015, observed differences between the groups (definite MD and controls) for all cVEMP parameters, and the abnormal responses were 3.00% absence of potential and 33.00% of abnormal latencies of the p13 and n23 waves.^21^

Regarding the means and limits of normality of the cVEMP parameters, there is great variability between the results (equipment, type of stimulus, adopted parameters). Therefore, it is important that each research center standardize the reference values based on exams performed in healthy individuals and in different age groups.^7^, ^10^

Studies suggest that the absence of cVEMP can be related to decreased sensitivity in the saccular region, occult vestibular alteration or insufficient muscle contraction.^9^ This last factor was ruled out in this study, since electromyography recordings to monitor the sternocleidomastoid muscle contraction were obtained. Other authors also inferred that, depending on the evolution of the disease, patients may show irreversible degeneration of the sensory epithelium of the saccular macula, culminating in the absence of the biphasic potential.^6^, ^12^, ^48^

Latency prolongation, especially of the first peak (p13), can be related to changes in the saccule such as high endolymphatic pressure, impairing sound transmission.^12^ Other investigators suggest other causes, such as vestibular-spinal tract lesions, such as multiple sclerosis, and a retrolabyrinthine abnormalitya.^36^, ^48^ The asymptomatic ears also showed increased wave latency, but to a lesser degree, indicating a possible bilateral occult alteration.^12^, ^21^

Inter-amplitude interval, a parameter that was reduced in the ear with MD in the present study, reflects the magnitude of the vestibular-spinal reflex.^10^ Many studies do not consider this parameter when it is assessed alone, since it has great intersubject variability, due to differences in muscle mass and tone of each individual.^9^, ^12^ For better control, the inter-amplitude interval in the present study was standardized by the electromyographic activity control. Some studies also found a decrease in peak-to-peak amplitude in patients with MD.^6^, ^37^

In turn, the AI in the current study did not exhibit a significant difference between the groups, and this result was attributed to the large standard deviation found in the CG, which translated into a high upper normative limit. A study by Silva et al.^21^ corroborates the findings of the present study and also found no difference in relation to AI between patients with MD and controls. However, several studies have reported abnormalities of this parameter in MD.^10^, ^11^ The increase in the asymmetry index can lead to two interpretations, one of which may be the amplitude increase in the affected ear, suggesting a high sensitivity of the saccular macula as the hydrops approaches the stapes footplate. However, most of the time, it reflects the decrease in the amplitude of the affected side compared to the healthy side, culminating in an elevated AI.^22^ Other studies have shown that the AI can help in the clinical monitoring of MD progression, as higher indexes were observed for more advanced disease stages, reflecting greater asymmetry between the labyrinths.^22^, ^36^

Studies in the literature suggest the possibility that cVEMP can predict involvement of the asymptomatic ear, either in cases of occult saccular hydrops or in alterations in the binaural interactions of the otolith-cervical reflex arc;^12^, ^21^ these are in harmony with the findings of the present study. cVEMP alterations in the asymptomatic ear are in line with the high prevalence of saccular hydrops and the high proportion of MD involvement in the contralateral ears.^5^, ^29^

The present study did not show a correlation of the test parameters with the duration of the disease. This result may be related to the small sample size and the large variation in the duration of the disease. As for the cVEMP, some studies did not find this correlation, either. A study by Osei-Lah et al.^36^ found no evidence between MD duration and the absence or presence of cVEMP. Katayama et al.,^49^ in turn, did not observe an association between the cVEMP results and the stage of the disease. As for the vHIT, the study by Cerchiai et al.,^50^ compared two groups of unilateral MD subjects, with one group treated with intratympanic gentamicin and the other group undergoing conservative therapy, and did not show a correlation with duration of the disease for either group. However, the study by Zulueta-Santos et al.,^19^ assessed 36 patients with definite MD, without a control group, and observed that as the disease progresses, the number of abnormalities in the six semicircular canals increased, more in the ipsilateral side and, particularly, in the posterior canals.

## Conclusion

The vHIT examination in MD showed decreased OVR gain for the lateral canals, corrective saccades in the lateral canal only, predominantly of the overt type and did not find any significant differences between the symptomatic and asymptomatic ears.

For cVEMP, the patients with MD showed absence of biphasic potential and latency prolongation, and a significant difference was observed between groups for the inter-amplitude parameter, both for symptomatic and asymptomatic ears. There was no correlation between the test results and the duration of disease.

## Conflicts of interest

The authors declare no conflicts of interest.
